# A panel of six immune-related mRNAs as biomarkers for tuberculosis diagnosis

**DOI:** 10.3389/fgene.2025.1544007

**Published:** 2025-03-20

**Authors:** Yutong Wei, Zilu Wen, Qinghua Xue, Lin Wang, Hui Chen, Lei Shi, Laiyi Wan, Leilei Li, Hongwei Li, Wentao Hao, Shulin Zhang, Ka-Wing Wong, Xiaoli Yu, Yanzheng Song

**Affiliations:** ^1^ School of Life Science and Technology, Wuhan Polytechnic University, Wuhan, China; ^2^ Department of Scientific Research, Shanghai Public Health Clinical Center, Fudan University, Shanghai, China; ^3^ Department of Thoracic Surgery, Shanghai Public Health Clinical Center, Fudan University, Shanghai, China

**Keywords:** tuberculosis, miRNA, immune gene signature, diagnosis, LASSO regression

## Abstract

**Objective:**

This study aims to screen common immunological markers of lung tissues and blood for diagnosis of tuberculosis (TB).

**Methods:**

Differentially expressed miRNAs (DEmRs) and mRNAs (DEGs) were obtained by whole-transcriptome sequencing profiles on 18F-FDG PET/CT high and low metabolic active regions in lung tissues of nine TB patients. Common miRNAs were screened by intersecting with DEmRs, four miRNA GEO datasets, and their target mRNAs were predicted through the miRTarbase and Tarbase databases. Then these mRNAs were intersected with DEGs, mRNAs from blood samples and immune-related genes, to construct a miRNA-mRNA interaction network, and the hub genes were identified by Cytoscape. The relationship between immune infiltration and hub genes were evaluated using Cibersort. Finally, a diagnostic model based on Lasso regression analysis was established and validated by qRT-PCR.

**Results:**

Five common miRNAs were obtained in both blood and tissues. Six immune-related mRNAs (*NEDD4*, *PLTP*, *RNASEL*, *SEMA7A*, *TAPBP*, and *THBS1*) were screened out. A diagnostic model was established and validated in the blood samples of 30 pairs (TB/health volunteers). The AUC for the 6-mRNA combination was 0.79.

**Conclusion:**

We screened six mRNAs as a combination for diagnosing tuberculosis.

## 1 Introduction

*Mycobacterium tuberculosis* (*Mtb*) is the causative agent of TB and primarily spreads through the air, infecting nearly all tissues and organs throughout the body, with tuberculosis being the most common form. According to WHO Global TB Report 2024, there were 10.8 million new TB cases and 1.25 million deaths globally in 2023. Additionally, the impact of the COVID-19 pandemic has led to sustained high TB mortality rates in recent years (World Health Organization, 2024). It is estimated that at least 2 billion people (25%–30% of the global population) are carriers of latent tuberculous infections (LTBI) (Seeberg, 2023). The immune response in TB differs between LTBI and active tuberculosis (ATB) which is contagious (Barry et al., 2009). Therefore, it is crucial for early and accurate diagnosis of ATB to enhance patients care, improve outcomes, and delay the transmission cycle of *Mtb*. The current methods for detecting TB involve culturing the microorganism, which requires 3–12 weeks (Rohner et al., 1997), Acid-Fast Bacilli (AFB) Smear Microscopy and the gamma-interferon release assay (IGRA). However, sputum smear testing has low sensitivity, while IGRA is complex, time-consuming, and expensive (Goletti et al., 2022; Nogueira et al., 2022). Although GeneXpert *MTB*/RIF Ultra demonstrates high sensitivity in the diagnosis of tuberculosis, it is a high-cost testing method (Quan et al., 2024). Therefore, there is an urgent need to identify cost-effective and rapid biomarkers for the diagnosis of tuberculosis.

miRNAs are small non-coding RNAs that finely regulate post-transcriptional gene expression through inhibiting mRNA transcription promoting their degradation (Bao et al., 2021). In mammals, it is estimated that over 60% of mRNAs are regulated by miRNAs (Friedman et al., 2009). This imbalance between mRNA and miRNA is associated with various pathological processes, including cancer, neurodegenerative diseases, and cardiovascular diseases (Abbas and Gaye, 2025; Kandettu et al., 2025; Liao et al., 2025). Recently miRNAs have received much attention for their vital roles in TB pathogenesis, particularly in modulating T cells, macrophages and cytokines during *Mtb* infection (Doghish et al., 2025). For instance, miR-224-5p, miR-324-5p, and miR-488-5p regulate the expression of the common target gene *CTLA4*, promoting TB-associated macrophage polarization (Huang et al., 2020). miRNA-4687-5p downregulated *NRAMP1* and effected *Mtb* survival in A549 cells, indicating its potential as a therapeutic targets (Meng et al., 2024). Specific expression patterns of miRNAs in body fluids (e.g., aberrant expression of miR-22, miR-20, miR-146a, miR-191, and miR-320) serve as potential markers of active tuberculosis versus latent infection and correlate with disease severity (Wang et al., 2022). Compared to our previous study about LncRNA (Wang et al., 2021), miRNAs exhibit higher specificity and multi-target regulation ability, which make them have higher potential as biomarkers for diagnosing tuberculosis.

The immune microenvironment of lung tissue reflects the specific immune responses to pulmonary infection with *Mtb* (Wang et al., 2023). However, obtaining lung tissue is challenging, and patients must meet surgical criteria. By this stage, the majority of the patients have already experienced significant disease progression, complicating treatment efforts. Meanwhile, the profiles of miRNAs and mRNAs in the TB lung tissues and blood are different, but have some relevance (Liu et al., 2018; Huang et al., 2021). Therefore, we needed to identify their common miRNAs and their target genes for effective and rapid diagnosis. By comparative analysis of transcriptome sequencing profiles in TB lung tissues and public databases, we aimed to screen for potential miRNAs and their target genes that could serve as rapid and accurate biomarkers for the diagnosis of tuberculosis.

## 2 Materials and methods

### 2.1 Data collection

In a previous study, our research group collected lung tissues samples from nine patients diagnosed with pulmonary tuberculosis (Supplementary Table S1), including nine samples with high metabolic activity (PET-high) and nine samples with low metabolic activity (PET-low) as identified by 18F-fluorodeoxyglucose positron emission tomography/computed tomography (18F-FDG-PET/CT) imaging, for RNA-seq analysis. The sequencing datasets were GSE276819, GSE158767, and GSE277481. The RNA extraction and whole transcriptome sequencing were performed using the same methods as our previous work (Wang et al., 2021). Besides, we collected whole blood samples from 30 TB patients and 30 healthy volunteers (Supplementary Table S2).

### 2.2 Analysis of miRNA and mRNA differential genes

The raw data in FASTQ format were processed with Trimmomatic (v0.30; https://github.com/usadellab/Trimmomatic) to filter out redundant and low-quality data (Supplementary Table S3). mRNA sequences reads were aligned to the reference genome using HISAT2 (v2.0.1; https://daehwankimlab.github.io/hisat2/), and gene expression levels were calculated using StringTie (v1.3.5; https://github.com/gpertea/stringtie). miRNAs were identified and their expression was evaluated using MiRDeep2 (v0.0.8; https://github.com/rajewsky-lab/mirdeep2). Differential expression analysis was performed with the DEGSeq Bioconductor package (v1.42.0; https://bioconductor.org/). The thresholds for significant differential expression of miRNA and mRNA were set as |log2FC|>1, p-value<0.05, tpm_FH > 1, and tpm_FL > 1, resulting in the identification of DEGs and DEmRs respectively.

### 2.3 Download datasets

From the Gene Expression Omnibus (GEO) database (https://www.ncbi.nlm.nih.gov/geo/): four miRNA datasets from blood samples were downloaded: GSE25435, GSE131174, GSE149645, and GSE229020; four mRNA datasets from blood samples were downloaded: GSE19491, GSE28623, GSE73408, and GSE101705 (Supplementary Table S4).

### 2.4 Construction of the miRNA-mRNA network

The differentially expressed genes from four miRNA datasets of the blood samples were intersected with DEmRs to obtain a refined set of miRNAs. These miRNAs were then uploaded to the miRTarbase (https://mirtarbase.cuhk.edu.cn/php/index.php) and Tarbase (http://carolina.imis.athenainnovation) to predicted target mRNAs. The predicted target mRNAs were further intersected with DEGs, four mRNA datasets of the blood samples, and immune-related genes obtained from the ImmPort Portal database (https://www.immport.org/). This process yielded the final miRNA-mRNA pairs.

Pearson correlation coefficients for miRNA-mRNA interactions were calculated based on the gene expression matrix. miRNA-mRNA pairs with |R|>0.4 and p-value<0.05 were selected, and the network was visualized using Cytoscape (V3.9.1; https://cytoscape.org/).

### 2.5 Functional enrichment analysis of mRNAs

To further investigate the biological mechanisms underlying potential biomarkers, we employed the clusterProfiler package (v3.16.1; https://www.bioconductor.org/packages/release/bioc/html/clusterProfiler.html) to perform Kyoto Encyclopedia of Genes and Genomes (KEGG) and Gene Ontology (GO) analyses on the differentially expressed genes. The parameters used for the analysis were as follows: minGSSize = 10, maxGSSize = 500, and p-value<0.05.

### 2.6 Immune infiltration analysis

Using the gene expression signature matrix of 22 immune cell subtypes (LM22) in the CIBERSORT (v0.1.0; https://github.com/Moonerss/CIBERSORT) package as the reference gene set, the immune infiltration analysis was performed using the cibersort function with the following parameters: perm = 1000, QN = T. The correlation coefficients and p-values between the abundance of immune cells and the expression levels of target genes were calculated using the cor function.

### 2.7 Construction of a lasso regression model

The Lasso regression analysis was conducted using the glmnet package in R (v4.1.8; https://cran.r-project.org/web/packages/glmnet/index.html) to perform binary logistic regression on gene expression levels. The coefficients of the regression model were extracted using the coef function to construct the regression model. The classification performance of the model was validated using the pROC package (v1.18.5; https://www.expasy.org/resources/proc) on the GEO dataset.

### 2.8 Quantitative reverse-transcription polymerase chain reaction (qRT-PCR) assay

The total RNA was extracted from blood using the QIAamp RNA Blood Mini Kit (Qiagen), and cDNA was synthesized from RNA using HiScript III RT SuperMix for qPCR (+gDNA wiper) (Vazyme) and amplified using the ChamQ Universal SYBR qPCR Master Mix (Vazyme). Subsequently, target gene was normalized to Hypoxanthine Phosphoribosyltransferase 1(*HPRT1*) and the relative expression levels were calculated with the 2^−ΔΔCt^ method. The specificity of the PCR products was evaluated using melt curve analysis. Real-time PCR was conducted using primers for *NEDD4*, *PLTP*, *RNASEL*, *SEMA7A*, *TAPBP*, and *THBS1*. The qRT-PCR primers were purchased from GeneWiz (Suzhou, China), and the primer sequences were listed in the Supplementary Table S6.

### 2.9 Statistical analysis

Statistical analysis of the experimental data was performed using GraphPad Prism software (version 9.5). The Student’s t-test was employed to compare continuous data. The accuracy of the 6-mRNA diagnostic method was evaluated using SPSS software (version 27.0.1). *p < 0.05 indicated statistical significance.

## 3 Results

**FIGURE 1:**
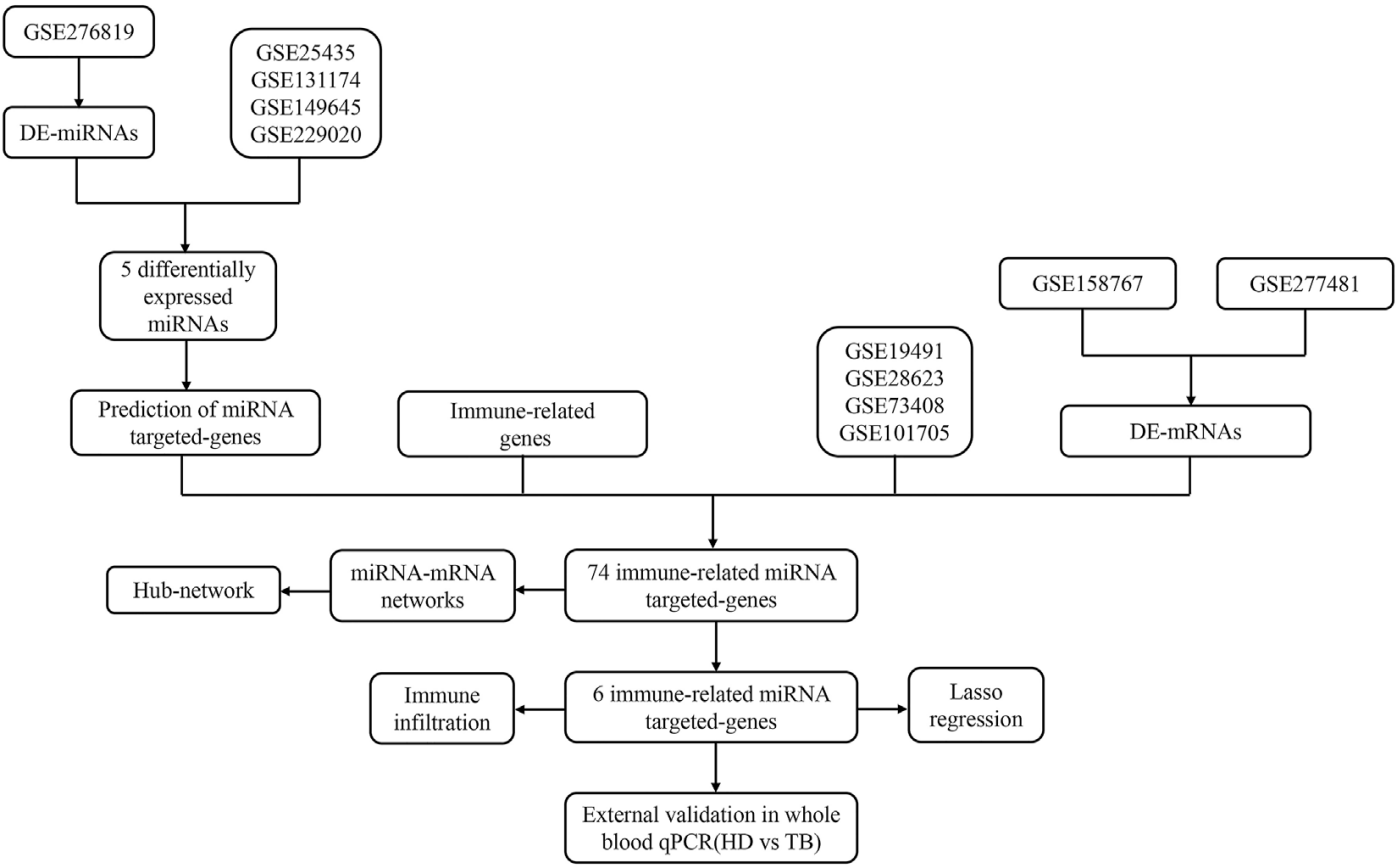
The flow chat of study.

The flow chart of this study was shown in Figure 1.

### 3.1 Differentially expressed miRNAs and mRNAs in TB lung tissues

**FIGURE 2:**
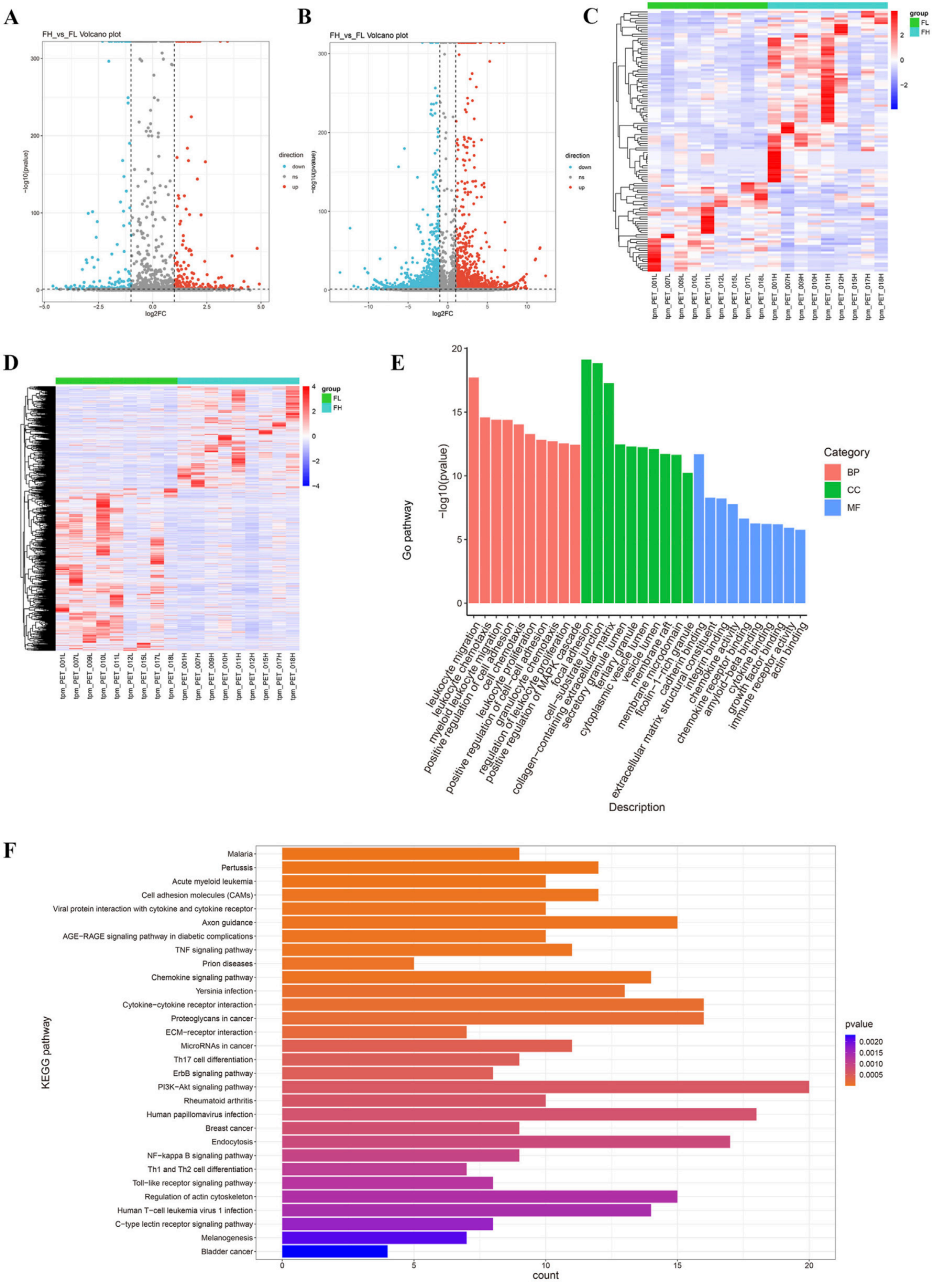
Differentially expressed miRNAs and mRNAs in TB lung tissues. **(A, B)** The volcano plots display DEmRs **(A)** and DEGs **(B)**; **(C, D)** The heatmaps illustrate the expression levels of DEmRs and DEGs, with the criteria of |log2FC|>1 & p-value<0.05 & tpm_FH > 1 & tpm_FL > 1; **(E)** The top 30 Gene Ontology (GO) terms enriched from DEGs, categorized into biological processes (BP), cellular components (CC), and molecular functions (MF); **(F)** KEGG pathway enrichment analysis of the top 30 enriched DEGs.

To understand the expression patterns of miRNA and mRNA in the lung tissues of tuberculosis patients, we performed a differential analysis of miRNA and mRNA between PET-high and PET-low lung tissues in the affected areas (Figures 2A, B). The basic criteria for screening DEGs were set as |log2FC|>1 & p-value<0.05 & tpm_FH > 1 & tpm_FL > 1. A total of 117 DEmRs were identified, with 77 miRNAs being upregulated and 40 miRNAs downregulated. In addition, 2014 DEGs were identified, including 1235 mRNAs that were upregulated and 779 mRNAs that were downregulated. Hierarchical clustering analysis of DEmRs and DEGs revealed a distinct distribution in PET-high and PET-low samples (Figures 2C, D).

GO functional enrichment analysis indicated that DEGs were significantly associated with leukocyte migration, focal adhesion, cell-matrix adhesion, collagen, and cadherins (Figure 2E). KEGG enrichment analysis demonstrated that DEGs were primarily involved in the interactions between cell adhesion molecules, viral proteins, cytokines, and cytokine receptors (Figure 2F).

### 3.2 Screening of miRNAs and construction of miRNA-mRNA network

**FIGURE 3:**
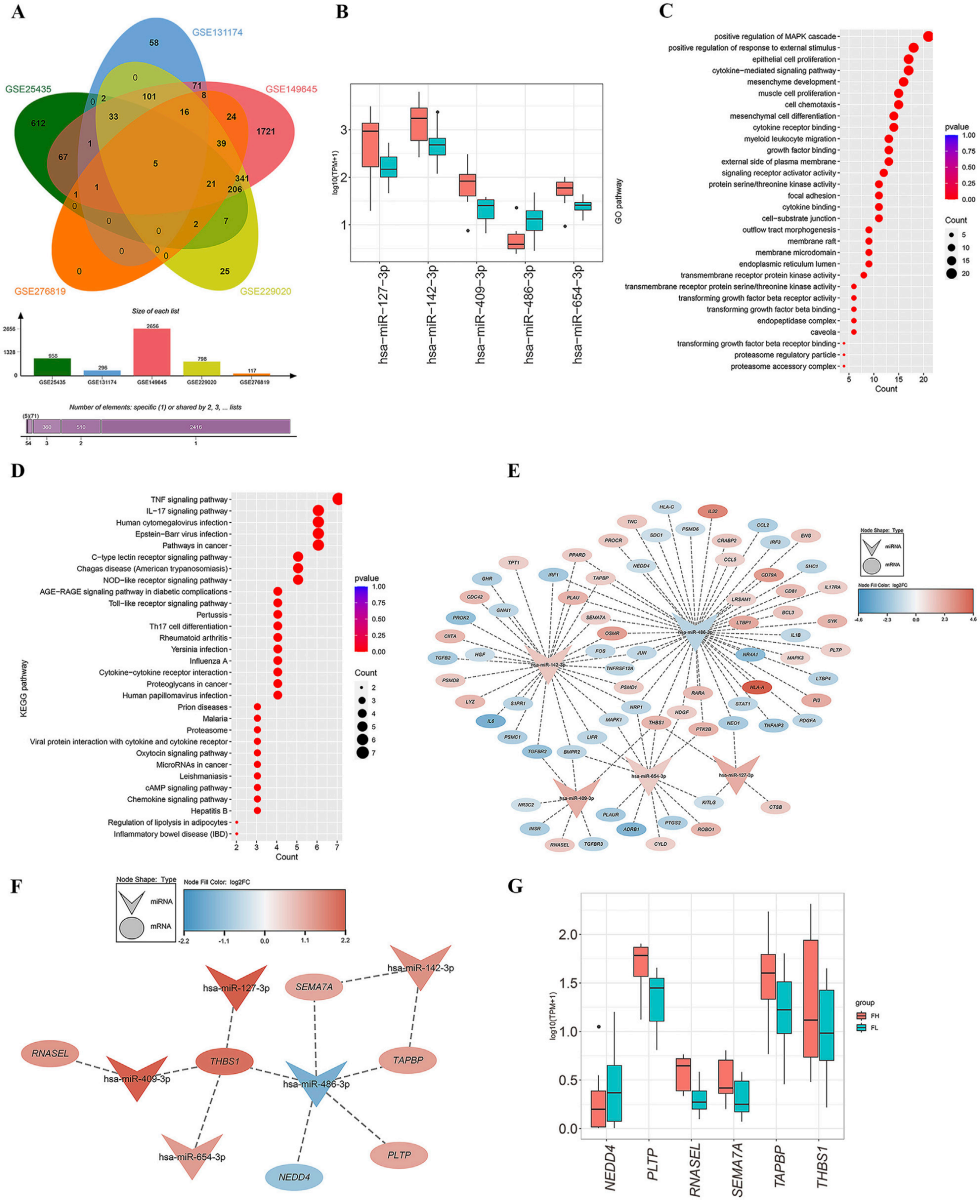
Screening of miRNAs and construction of miRNA-mRNA network. **(A)** The Venn diagram demonstrates the overlapping genes between DEmRs from lung tissues RNA-seq and the blood datasets GSE131174, GSE14964, GSE229020 and GSE25435. **(B)** Expression levels of the five differentially expressed miRNAs in PET-high/low lung tissues. **(C, D)** The top 30 Gene Ontology (GO) terms in biological processes (BP), cellular components (CC), and molecular functions (MF) for the targeted mRNAs of the miRNAs **(C)**, as well as the top 30 enriched pathways of differentially expressed mRNAs in the KEGG analysis **(D)**. **(E)** The miRNA-mRNA co-expression network constructed from differentially expressed miRNAs and their predicted target genes. **(F)** Subnetwork diagram of the miRNA-mRNA network with |R|>0.4 and P < 0.05. **(G)** Expression levels of six mRNAs in PET-high and PET-low lung tissues.

We hypothesized that miRNAs associated with tuberculosis infection in lung tissues could also be detected in blood samples. Consequently, we screened for the overlapping genes between DEmRs and the miRNA datasets GSE131174, GSE14964, GSE229020, and GSE25435 from blood samples (Figure 3A), and obtained five differentially expressed miRNAs: has-miR-409-3p, has-miR-486-3p, has-miR-127-3p, has-miR-654-3p, and has-miR-142-3p (Supplementary Table S5). Besides, we found that has-miR-486-3p was downregulated in PET-high tissues, while the other four miRNAs showed significantly higher expression levels compared to the PET-low tissue samples (Figure 3B).

Furthermore, these miRNAs were uploaded to the miRTarbase and Tarbase databases for summarizing all predicted target genes. Subsequently, by screening for overlapping genes between the miRNA target genes, four datasets of blood samples and an RNA-seq dataset from lung tissues, as well as an immune-related gene set downloaded from the ImmPort Portal database, a final set of 74 immune-related target genes were obtained (38 genes downregulated and 36 genes upregulated). GO enrichment analysis of these 74 targeted mRNAs revealed significant enrichment in biological processes (BP) such as positive regulation of MAPK cascade, positive regulation of response to external stimulus, epithelial cell proliferation, and cytokine-mediated signaling pathways (Figure 3C). Concurrently, KEGG enrichment analysis showed that the differentially expressed genes were primarily involved in the TNF signaling pathway, IL-17 signaling pathway, human cytomegalovirus infection, Epstein-Barr virus infection, and cancer-related pathways (Figure 3D).

Based on the gene expression matrix, we generated a heatmap and calculated the Pearson correlation coefficients for miRNA-mRNA interactions (Supplementary Figure S1). The criteria for selecting miRNA-mRNA pairs were set as |R|>0.4 and p < 0.05. Then, we used Cytoscape to perform a detailed analysis of the selected 74 overlapping genes and constructed a miRNA-mRNA co-expression network (Figure 3E). Ultimately, we determined six miRNA target genes: *NEDD4*, *PLTP*, *RNASEL*, *SEMA7A*, *TAPBP*, and *THBS1* (Supplementary Table S5). The miRNA-mRNA network analysis revealed that has-miR-486-3p acted as a sponge for the key gene *NEDD4*; has-miR-142-3p regulated the expression of target genes *TAPBP* and *SEMA7A*; the expression of *RNASEL* was regulated by has-miR-409-3p; and the four miRNAs, has-miR-409-3p, has-miR-127-3p, has-miR-486-3p, and has-miR-654-3p, may co-regulate the target gene *THBS1* (Figure 3F). Notably, the genes *PLTP*, *RNASEL*, *SEMA7A*, *TAPBP*, and *THBS1* exhibited high expression levels in PET-high tissue samples, while *NEDD4* was found to be downregulated (Figure 3G).

### 3.3 Relationship between miRNA target-genes expression and immune infiltration

**FIGURE 4:**
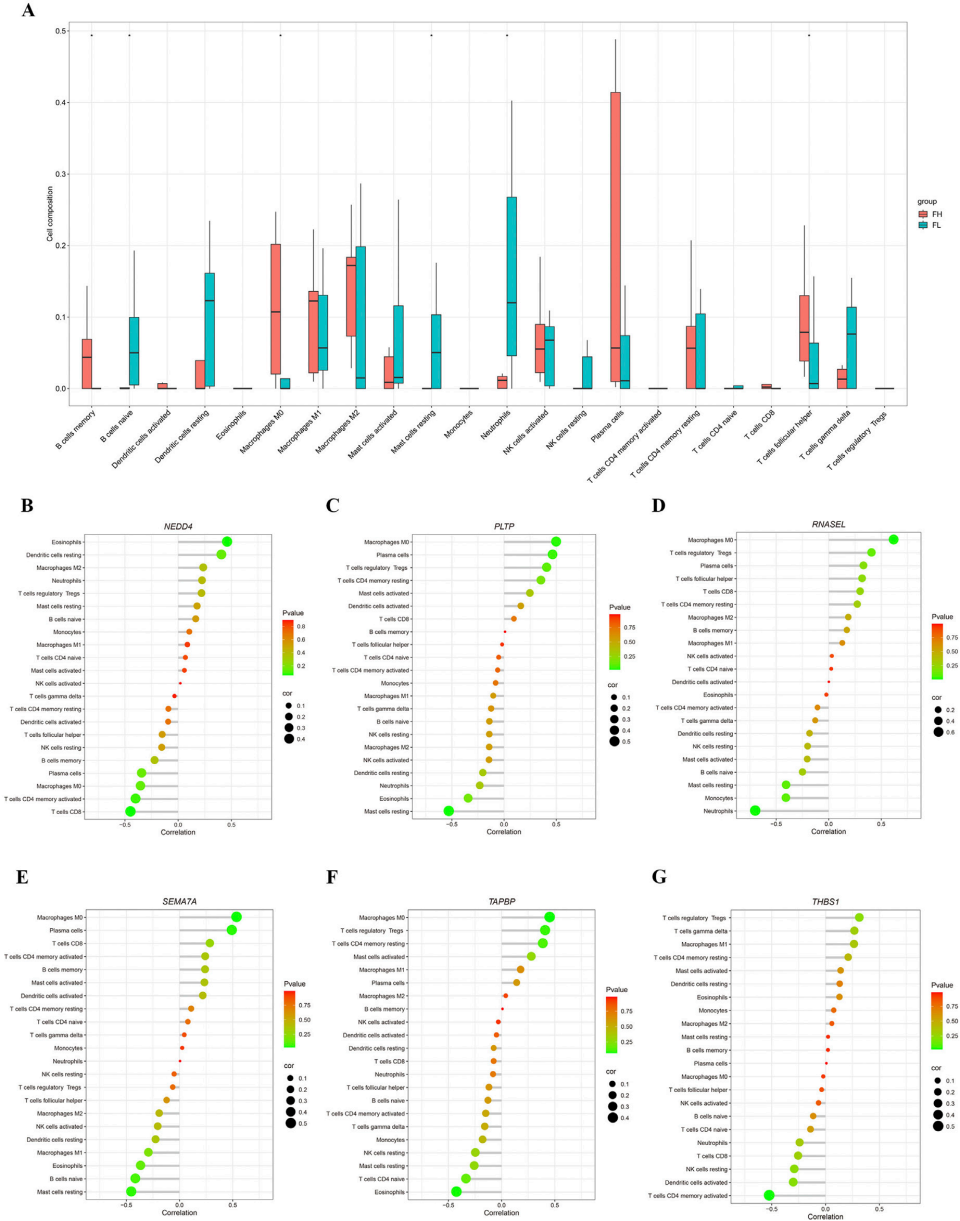
Analysis of immune infiltration. **(A)** Correlation PET/CT-high/low tuberculosis tissues samples with immune infiltration level. **(B–G)** Correlation of *NEDD4*, *PLTP*, *RNASEL*, *SEMA7A*, *TAPBP*, and *THBS1* expressions with immune infiltration level.

Further we explored the potential connections between the six miRNA target genes and immune cells. Using the gene expression signature matrix of 22 immune cell subtypes (LM22) from the CIBERSORT algorithm as a reference, we analyzed and compared the immune cell composition between PET-high and PET-low (control) tissues. As showed, the proportion of M2 macrophages significantly increased in PET-high tissues compared to PET-low tissues, while the proportion of neutrophils notably decreased (Figure 4A). Then the expression changes of the six selected immune-related genes were analyzed, and the results showed that the hub genes, *PLTP* (Figure 4C), *RNASEL* (Figure 4D), *SEMA7A* (Figure 4E), and *TAPBP* (Figure 4F) exhibited a positive correlation with the infiltration states of M0 macrophages and plasma cells, while showing a negative correlation with the resting states of neutrophils, mast cells, eosinophils and NK cells. Additionally, the hub genes *NEDD4* (Figure 4B) and *THBS1* (Figure 4G) was found to be positively correlated with the infiltration states of eosinophils and Treg cells, but negatively correlated with the resting infiltration of CD4^+^ memory T cells, CD8^+^ T cells, and NK cells.

### 3.4 Construction of a diagnostic model

**FIGURE 5:**
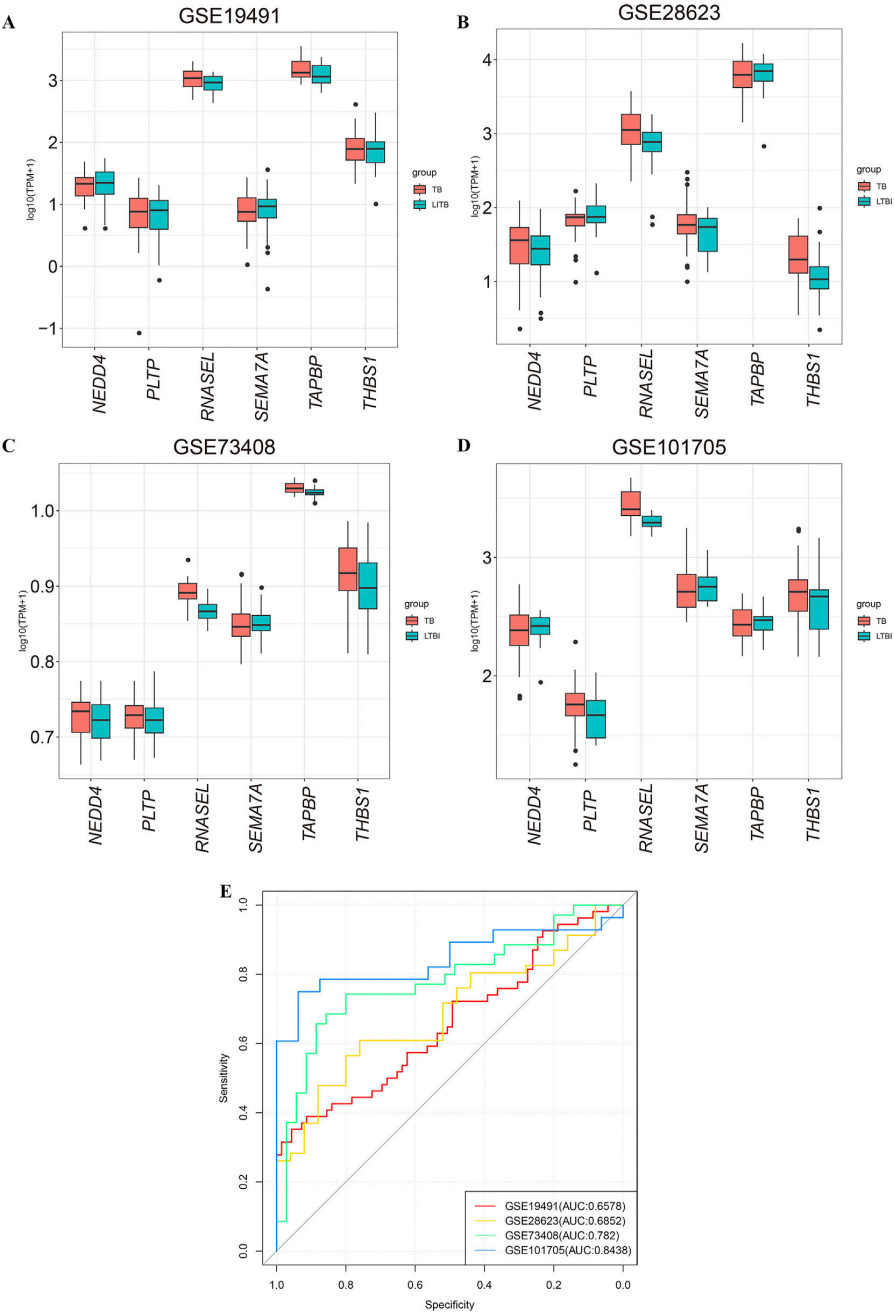
Validation of the expression of six mRNAs in GEO datasets. **(A–D)** The expression of six candidate miRNA targeted-genes in GSE19491, GSE28623, GSE73408, and GSE101705. **(E)** ROC curves for diagnosing tuberculosis using the 6-mRNA signature. The AUC indicates that the 6-mRNA signature demonstrates good predictive performance across the datasets GSE19491, GSE28623, GSE73408 and GSE101705. LTBI: latent tuberculosis infection; TB: tuberculosis.

To validate the significance of the six selected mRNAs, we assessed their expression in four independent datasets (GSE19491, GSE28623, GSE73408, and GSE101705), which include samples from LTBI and TB (Figures 5A–D). *NEDD4*, *PLTP*, *RNASEL*, *SEMA7A*, *TAPBP*, and *THBS1* were combined into a 6-mRNA feature panel for diagnosis of tuberculosis. Use lasso regression in the logistic regression model fitting data, the model in the form of: logit (p)/(1 - p) = β_0_+ β_1_β_2_+β_2_β_2_+…+β_n_ X_n._ Where p is the probability of TB, X1, X2, … Xn is the gene expression level, β_0_, β_1_, … , β_n_ is the model parameter. The probability equation for predicting TB was as follows: 6-mRNA = NEDD4*-0.117407108+PLTP*0.00241744+RNASEL*1.097231674+SEMA7A*0.727327735+TAPBP*-0.004076635+THBS1*0.028823439. The six genes are predictor variables for gene expression levels, and the coefficient in front of each variable represents the effect of that gene expression level on the log-odds of developing tuberculosis disease.

The area under the curve (AUC) for the datasets GSE19491, GSE28623, GSE73408, and GSE101705 were 0.6578, 0.6852, 0.782 and 0.8438 respectively (Figure 5E). The above results suggested the combination of the 6-mRNA features in the validation cohort demonstrated significant diagnostic efficacy.

### 3.5 Validation of 6-mRNAs combination signature in blood samples

**FIGURE 6:**
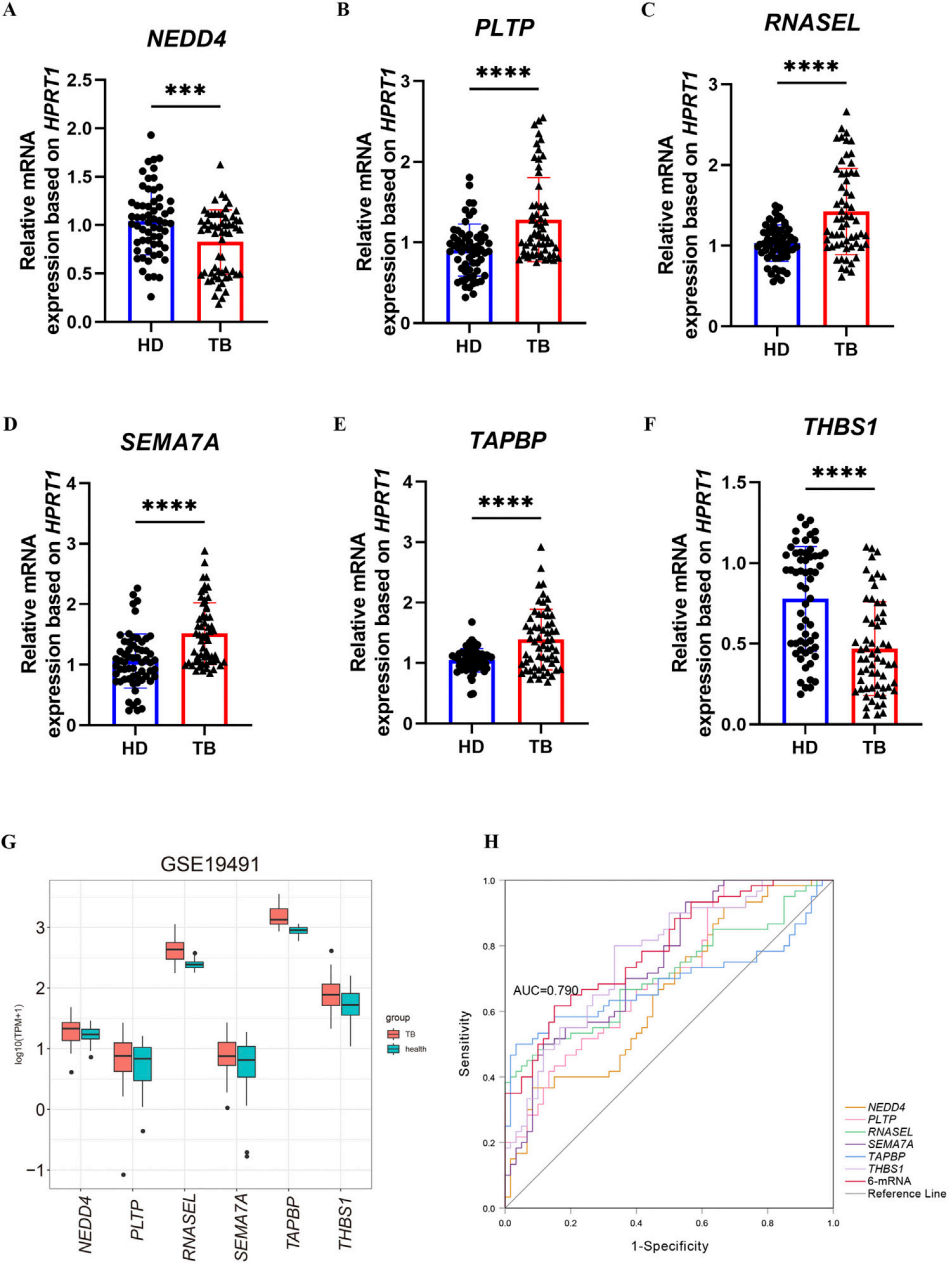
Validation of the expression of six mRNAs in blood samples. **(A–F)** The expression levels of *NEDD4*, *PLTP*, *RNASEL*, *SEMA7A*, *TAPBP* and *THBS1* were confirmed using qRT-PCR in health and TB whole blood. **(G)** validation of mRNA by GSE19491. **(H)** ROC for diagnosing TB by the 6-mRNA signature between patients and health in the combined or respective mRNA. The p-value were calculated using Student’s test. ***p < 0.001, ****p < 0.0001.

Tissue samples are advantageous for identification, whereas liquid samples such as blood are more suited for diagnosis, prognosis, and disease detection. We collected whole blood samples from 30 patients with pulmonary tuberculosis and 30 healthy volunteers to validate the expression of six selected mRNAs previously through qRT-PCR. The results showed that *PLTP* (Figure 6B), *RNASEL* (Figure 6C), *SEMA7A* (Figure 6D), and *TAPBP* (Figure 6E) were upregulated in the whole blood of tuberculosis patients, while *NEDD4* (Figure 6A) and *THBS1* (Figure 6F) were downregulated. We also assessed the expression levels of these six mRNAs in the GSE19491 dataset (Figure 6G), and found that the results for *PLTP*, *RNASEL*, *SEMA7A*, and *TAPBP* were consisted with the results of qRT-PCR. The receiver operating characteristic (ROC) curve analysis for the 6-mRNA signature revealed an AUC of 0.790, with a 95% confidence interval (CI) ranging from 0.712 to 0.869 (Figure 6H).

## 4 Discussion

More than half of the suspected cases of tuberculosis worldwide remain undiagnosed, highlighting complexity of its diagnosis (Global Tuberculosis Report 2024 Factsheet, n. d.). Studies have shown that differentially expressed miRNAs play a crucial role in tuberculosis by regulating the interactions between *Mtb* and the host (Silva et al., 2021). In this study, we aimed to identify immune cell-related biomarkers by exploring miRNA-mRNA interactions.

This study analyzed the differentially expressed miRNAs and mRNAs in lung tissues with high/low metabolic activity as identified by PET/CT in patients undergoing surgical resection for pulmonary tuberculosis. By comparing these findings with tuberculosis-related blood databases, we identified five miRNAs (has-miR-409-3p, has-miR-127-3p, has-miR-486-3p, has-miR-654-3p, and has-miR-142-3p). Among these, has-miR-486-3p plays a crucial role in the regulation of granuloma formation in tuberculosis and its associated physiopathology (Silva et al., 2021). Additionally, has-miR-127-3p promotes disease progression by targeting *JAK1* to activate the type I interferon signaling pathway (Wu et al., 2021).

Consequently, we identified corresponding target genes in the bloodstream based on differentially expressed miRNAs in the lung tissues of tuberculosis patients and investigated whether these target genes could serve as biomarkers for the diagnosis of tuberculosis. By taking the intersection of these target genes of these miRNAs with the blood databases and immune-related gene sets, the Pearson correlation coefficient was calculated, six key genes (*NEDD4*, *PLTP*, *RNASEL*, *SEMA7A*, *TAPBP*, and *THBS1*) were selected. Furthermore, miRNA-mRNA network analysis revealed the regulatory relationships between specific miRNAs and their target genes.

Previous studies have shown that immune infiltration influences the progression of tuberculosis. Therefore, we utilized the CIBERSORT algorithm to analyze the relationship between the six target genes and immune infiltration. As expected, *NEDD4*, *PLTP*, *RNASEL*, *SEMA7A*, *TAPBP*, and *THBS1* were associated with immune cell states. We further examined the expression levels of these genes in PET-high and PET-low lung tissues from tuberculosis patients. The results showed significant differences in expression and notably, most genes were upregulated in PET-high tissues compared to PET-low, while *NEDD4* was downregulated. The validation in whole blood also confirmed that, the qPCR results for these hub genes, except *THBS1*, were consistent with the bioinformatics findings.

NEDD4 is an E3 ubiquitin ligase that has recently been linked to inflammatory responses and oxidative stress, playing a crucial role in the pathogenesis of pulmonary diseases (Cockram et al., 2021). It may mitigate pulmonary fibrosis by mediating the ubiquitination of YY1 (Chen et al., 2024). The miRNA-mRNA network indicated that *NEDD4* was negatively regulated by has-miR-486-3p, resulting in decreased expression levels. This finding aligned with our validation results, which showed reduced expression levels of *NEDD4* in the blood of tuberculosis patients (Figure 6A).

Phospholipid transfer protein (PLTP), a widely expressed key lipid transfer protein, not only influences the transport of plasma triglycerides and cholesterol but also responds to pro-inflammatory stimuli and exhibits anti-cancer properties (Schlitt et al., 2005). Moreover, *PLTP* has been shown to inhibit neutrophil degranulation (Ochieng et al., 2018). Increased expression of *PLTP* has been observed in the lung tissues of patients with chronic obstructive pulmonary disease (COPD) (Jiang et al., 1998). Based on these findings, we hypothesized that *PLTP* played a crucial role in lipid metabolism and immune cell recruitment in tuberculosis.

*RNASEL*, a crucial ribosome-related gene, plays an essential role in various cellular physiological processes. Studies have demonstrated that RNase L is necessary for the induction of pro-inflammatory cytokines and regulates lysosomal enzyme activity, thereby facilitating the digestion of bacteria (Li et al., 2008). Additionally, it mediates antibacterial activity through apoptosis (Castelli et al., 1997). Our analysis of clinical samples from tuberculosis patients revealed a significant increase in *RNASEL* expression in the serum of patients with pulmonary tuberculosis compared to healthy volunteers (Figure 6C).

Semaphorin 7A (SEMA7A), a member of the “immune” signaling family, stimulates macrophages to produce pro-inflammatory factors through β1 integrin, initiating the inflammatory response (Suzuki et al., 2007). In patients with idiopathic pulmonary fibrosis (IPF), the expression of *SEMA7A* on CD4^+^CD25+FoxP3+ regulatory T cells (Tregs) is associated with disease progression (Reilkoff et al., 2013). We found that *SEMA7A* expression was upregulated in both lung tissues and blood samples of tuberculosis patients (Figures 3G, 6D). The upregulation of *SEMA7A* may exacerbate pulmonary fibrosis and reflect disease severity, suggesting its potential as a diagnostic marker for pulmonary tuberculosis.

*TAPBP* encodes a transporter associated with antigen processing (TAP) binding protein, also known as Tapasin (Bach et al., 1997). As a component of the peptide-loading complex, Tapasin plays a crucial role in selecting high-affinity peptides for binding to MHC class I molecules (Howarth et al., 2004). Studies have shown that individuals with *TAPBP* gene mutations are more susceptible to bacterial infections, particularly from *Streptococcus pneumoniae* and *Haemophilus influenzae* (Yabe et al., 2002). In clinical samples from tuberculosis patients, the expression level of *TAPBP* is elevated, leading to enhanced antigen presentation and an increased proportion of CD8^+^ T cell immune infiltration (Figure 6E). However, our immune infiltration analysis revealed downregulation *TAPBP* gene within CD8^+^ T cells (Figure 4F). This may be attributed to either decreased expression or dysfunction of MHC class I molecules on CD8^+^ T cells, impairing antigen presentation and preventing CD8^+^ T cells from effectively recognizing and eliminating cells infected by *Mtb*. Furthermore, chronic inflammation caused by long-term pulmonary tuberculosis infection could lead to immune tolerance, thereby suppressing CD8^+^ T cell function while simultaneously increasing *TAPBP* expression levels.

Thrombospondin 1 (THBS1), a member of the platelet-reactive protein family, is a glycoprotein produced by various cells, including platelets and macrophages (Jefferson et al., 2020). Research has shown that *THBS1* inhibits endothelial nitric oxide synthase (eNOS) (Bauer et al., 2010), reducing nitric oxide (NO) production, which is considered an important molecule in the defense against *Mtb* (Braverman and Stanley, 2017). In our study using whole blood samples from tuberculosis patients, we found that *THBS1* expression was downregulated (Figure 6F). That is probably because *THBS1* can regulate vascular permeability (Bauer et al., 2010), and its decreased expression may lead to increased vascular permeability, further aggravating pulmonary edema and worsening lung disease. These findings suggest that *THBS1* is a promising candidate as both a novel therapeutic target and a potential biomarker for diagnosing tuberculosis.

The discovery of these six mRNAs (*NEDD4*, *PLTP*, *RNASEL*, *SEMA7A*, *TAPBP*, and *THBS1*) not only reveals critical molecular mechanisms underlying immune regulation in tuberculosis but also establishes a robust theoretical foundation for advancing diagnostic and therapeutic strategies. By integrating these genes into a diagnostic panel, we developed a Lasso regression model that effectively distinguishes tuberculosis patients from healthy individuals. This model demonstrates significant potential for screening and clinical application, particularly due to its cost-effectiveness, rapid turnaround time, and high diagnostic accuracy (AUC: 0.790 in qPCR validation).

Despite its advancements, this study still has some limitations, including reliance on surgical lung tissue, public datasets and a small validation cohort in the blood samples, which may limit generalizability. For further experiments, multi-center studies with diverse populations and mechanistic research are needed to validate the model and explore gene functions in tuberculosis pathogenesis. Additionally, we plan to integrate the model with existing diagnostic standards to achieve better diagnostic performance. In conclusion, this work provides a practical diagnostic tool, and lays the groundwork for improving diagnosis, treatment, and patient outcomes.

## Data Availability

The data presented in the study are deposited in the GEO database repository, accession numbers GSE158767, GSE276819, and GSE277481.
