# Supplementary material for: A panel of six immune-related mRNAs as biomarkers for tuberculosis diagnosis
Source: Front Genet. 2025 Mar 20;16:1544007. doi: 10.3389/fgene.2025.1544007 (PMC11965592; doi:10.3389/fgene.2025.1544007)
Supplement: Supplementary file 3 [file Table1.docx]

Supplementary Materials

**Supplementary Figure**


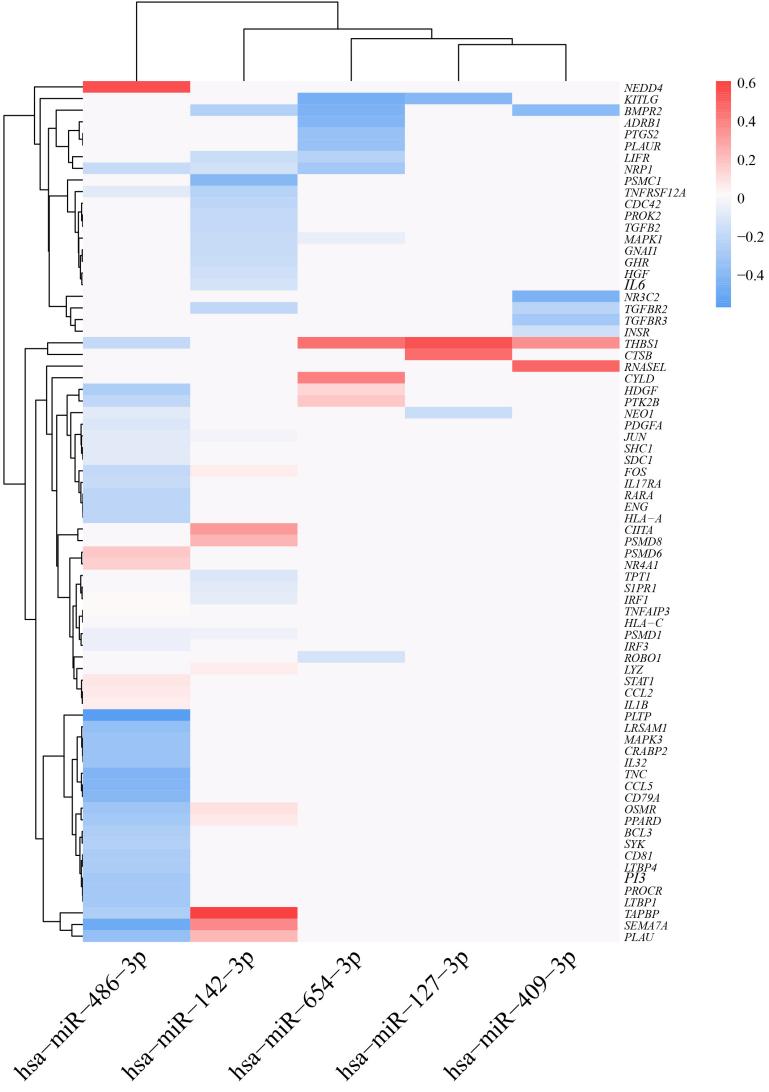


**Supplementary Figure 1**. Heatmap of the correlations between miRNAs and their targeted mRNAs

**Supplementary Tables**

**Supplementary Table 1.** Clinical information of transcriptome samples

**Supplementary Table 2.** Participants clinical information.

**Supplementary Table 3**. FL_vs_FH. differentially expressed miRNAs and mRNAs

**Supplementary Table 4.** Database information

| **Number** | **Type** | **Samples(LTB/ATB)** |
| --- | --- | --- |
| GSE158767 | Lung tissues | 0/9 |
| GSE276819 |  |  |
| GSE229020 | blood miRNAs | 6/12 |
| GSE149645 | blood miRNAs | 16/16 |
| GSE131174 | blood miRNAs | 21/8 |
| GSE25435 | blood miRNAs | 3/3 |
| GSE19491 | blood mRNAs | 69/54 |
| GSE28623 | blood mRNAs | 25/46 |
| GSE73408 | blood mRNAs | 35/35 |
| GSE101705 | blood mRNAs | 16/28 |

**Supplementary Table 5.** Differentially expressed miRNAs and mRNAs in lung tissues

| **miRNA** | **Log2FC** | **P value** | **adj P value** |
| --- | --- | --- | --- |
| has-miR-127-3p | 2.17 | <0.001 | <0.001 |
| has-miR-142-3p | 1.43 | <0.001 | <0.001 |
| has-miR-409-3p | 2.12 | <0.001 | <0.001 |
| has-miR-486-3p | -1.5 | <0.001 | <0.0014 |
| has-miR-654-3p | 1.29 | <0.001 | <0.001 |

| **mRNA** | **Log2FC** | **P value** | **adj P value** |
| --- | --- | --- | --- |
| *NEDD4* | -1.11 | 0.0076143610 | 0.140226259 |
| *PLTP* | 1.16 | <0.001 | <0.001 |
| *RNASEL* | 1.38 | 0.0057678900 | 0.114828858 |
| *SEMA7A* | 1.14 | 0.0276068740 | 0.337296024 |
| *TAPBP* | 1.24 | <0.001 | <0.001 |
| *THBS1* | 1.9 | <0.001 | <0.001 |

**Supplementary Table 6.** Oligonucleotide primer sets for qPCR

| **Name** | **Sequence(5’-3’)** | **Length** |
| --- | --- | --- |
| HPRT1-F | TTGCTGACCTGCTGGATTAC | 136 |
| HPRT1-R | TCTCCACCAATTACTTTTATGTCC |  |
| NEDD4-F | TGGAGAATTGGAAAGACCACAT | 262 |
| NEDD4-R | ACCTAGGAAATCATCTCTTGTCTTG |  |
| PLTP-F | AAGAACAACAGAAAGGGTGAAGG | 173 |
| PLTP-R | CTGAGGCGTTGATGTAGCCC |  |
| RNASEL-F | AGCATTTCCCCCAAACCCAC | 230 |
| RNASEL-R | ATACACGATGCCAGGGACTG |  |
| SEMA7A-F | CCGTCTGGAAAGGCCATGTA | 177 |
| SEMA7A-R | AGTTCTCGCAGTCCGTGC |  |
| TAPBP-F | CAGTGTACACGACCCCGC | 122 |
| TAPBP-R | TGGCCCATTTCGCAGAGG |  |
| THBS1-F | GCTGCGCCCGAGCTG | 157 |
| THBS1-R | CAGCAGGGATCCTGTGTGT |  |
